# Single-step co-deposition of nanostructured tungsten oxide supported gold nanoparticles using a gold–phosphine cluster complex as the gold precursor

**DOI:** 10.1088/1468-6996/15/6/065004

**Published:** 2014-12-09

**Authors:** Anara Molkenova, Rozie Sarip, Sanjay Sathasivam, Polona Umek, Stella Vallejos, Chris Blackman, Graeme Hogarth, Gopinathan Sankar

**Affiliations:** 1 Department of Chemistry, University College London, 20 Gordon Street, WC1H 0AJ London, UK; 2 Solid State Physics Department, Jožef Stefan Institute, Slovenia; 3 London Centre for Nanotechnology, 17–19 Gordon Street, WC1H 0AH London, UK; 4 Department of Chemical Engineering, Graduate School of Science and Engineering, Tokyo Institute of Technology, Japan; 5 SIX Research Center, Faculty of Electrical Engineering and Communication, Brno University of Technology, Technicka 12, CZ-61600 Brno, Czech Republic; 6 Department of Chemistry, King's College London,Britannia House, 7 Trinity Street, SE1 1DB London, UK

**Keywords:** CVD, nanoparticle, catalyst

## Abstract

The use of a molecular gold organometallic cluster in chemical vapour deposition is reported, and it is utilized, together with a tungsten oxide precursor, for the single-step co-deposition of (nanostructured) tungsten oxide supported gold nanoparticles (NPs). The deposited gold-NP and tungsten oxide supported gold-NP are highly active catalysts for benzyl alcohol oxidation; both show higher activity than SiO_2_ supported gold-NP synthesized via a solution-phase method, and tungsten oxide supported gold-NP show excellent selectivity for conversion to benzaldehyde.

## 1. Introduction

We report a vapour phase method for the one-step preparation of supported gold nanoparticles (NPs), achieved by *in situ* decomposition of a molecular gold cluster and an organometallic tungsten precursor, and on the catalytic properties of the co-deposited material. Vapour synthesis of gold NPs, e.g. via chemical vapour deposition (CVD), offers advantages over wet-chemical techniques; it circumvents several steps associated with solution methods such as washing, drying, calcination and reduction, and can avoid changes in metal dispersion that can occur during high temperature calcination or reduction steps [1]. The use of well-defined molecular precursors in CVD could potentially provide control of the particle size, a critical parameter for the reactivity of the NP [2], by controlling the number of atoms involved in the particle. However, large molecular precursors are expected to have limited volatility and are therefore unsuitable for use in traditional CVD methods; the use of aerosol assisted (AA)CVD, in which the precursor is delivered as an aerosol of a precursor solution rather than as a vapour, allows a wider range of precursors to be exploited as solubility is the key precursor requirement as opposed to volatility [3].

A variety of synthetic strategies to supported metal NP are described in the literature [4–7], however the use of single-step routes in which both support and catalytic metal are synthesized together are infrequently reported despite the advantage of fewer processing steps [8]. We have recently reported the successful use of AACVD for the one-step synthesis of metal NPs supported on tungsten oxide, using inorganic metal precursors (H_*x*_MCl_*y*_, where M = Au, *x* = 1, *y* = 4 or M = Pt, *x* = 2, *y* = 6) [9, 10]. One concern in using these inorganic precursors for synthesis of catalytic metal particles is potential for incorporation of chlorine, which can poison the catalyst. Non-halide containing mononuclear gold–phosphine complexes, for instance [R–Au–PR_3_] [11, 12] and [Me–Au–P(OMe)_2_R′] [13], are known CVD precursors for the deposition of gold films, and the molecular gold cluster [Au_9_(PPh_3_)_8_](NO_3_)_3_ has been used as a precursor to supported metallic Au particles via solution-phase impregnation [14], however the use of molecular gold clusters in CVD has not previously been reported.

## 2. Experimental details

[Au_9_(Ph_2_P(p-tol))_8_](NO_3_)_3_ was prepared as described in the literature [15], with slight modification. A solution of NaBH_4_ (0.0049 g, 0.119 mmol) in ethanol (10 cm^3^) was added to a suspension of Au(Ph_2_P(p*-*tol))NO_3_ (0.2522 g, 0.467 mmol) in ethanol (10 cm^3^). After all the starting material had reacted the solution was taken to dryness giving a brown solid (0.2060 g), which was extracted with tetrahydrofuran (10 cm^3^) and then filtered resulting in a dark green solid (78% yield based on gold). Nuclear magnetic resonance (NMR) analysis: ^1^H-NMR (MeOD) *δ* (ppm) 7.39−6.51(m); 2.10(s). ^31^P{^1^H}-NMR: *δ* (ppm) 56.56(s). Elemental analysis gives C, 40.13%; H, 2.97%; N, 2.41%; as Au_9_P_8_C_152_H_136_N_3_O_9_ requires C, 43.79%; H, 3.29%; N, 1.01%.

Gold NPs were deposited at 350 °C via AACVD of [Au_9_(PPh_2_(p-tol))_8_](NO_3_)_3_ (3.5 mg) dissolved in methanol (10 cm^3^, Sigma-Aldrich, ≥99.6%) and tungsten oxide nanostructures were deposited at 500 °C via AACVD of tungsten hexaphenoxide (75 mg, W(OPh)_6_) [16] dissolved in acetone (10 cm^3^, Sigma-Aldrich, ≥99.6%) both following the previously reported method [9]. For co-deposition reactions 3.5 mg [Au_9_(PPh_2_(p-tol))_8_](NO_3_)_3_ was added to 75 mg W(OPh)_6_ dissolved in acetone and deposition was conducted at 500 °C. Deposition time varied depending on the solvent system, between 45 and 60 min. Glass substrates (10 mm × 10 mm × 1 mm) were used and cleaned with acetone and propan-2-ol prior to use.

Coupled differential scanning calorimetry–thermal gravimetric analysis (DSC–TGA) was performed on the gold precursor using a Netzsch STA 449C instrument. The TGA was carried out at atmospheric pressure, under a flow of helium gas (50 cm^3^ min^−1^), and the rate of heating was 2 °C min^−1^. The morphology of the samples was examined using scanning electron microscopy (SEM—Jeol 6301F, 5 keV), the structure using x-ray diffraction (XRD—Bruker, AXD D8 Discover, using Cu K*α* radiation, operated at 40 KV and 40 mA) and the chemical composition using wavelength dispersive x-ray spectroscopy (WDX—Philips, XL30ESEM). Transmission electron microscopy (TEM; JEOL JEM-100CX II, 100 kV) and high resolution TEM (HRTEM—Jeol 2100, 200 keV) measurements were performed by removing the film from the substrate by sonication of the substrates in methanol. X-ray photoelectron spectroscopy (XPS) was carried out using a Thermo K-*α* spectrometer in constant analyser energy mode and monochromated Al K*α* radiation. Survey scans were collected over the 0–1400 eV binding energy range with 1 eV resolution and a pass energy of 200 eV. Higher resolution scans (0.1 eV) encompassing the principal peaks of W, O, Au, C, Si and P were also collected at a pass energy of 50 eV. Data was processed using CasaXPS.

Catalytic tests were performed on the samples on glass substrates using a Multimax Multiple Automatic Lab described previously [17]. The reaction mixture (4.5 cm^3^ benzyl alcohol (BDH Chemical), 4.5 cm^3^ dodecane (Acros organics, 99%), 11.4 cm^3^ tert-butyl hydroperoxide (Sigma-Aldrich, 5–6 M solution in decane)) was heated under reflux (94 ± 1 °C) for 6 h and liquid samples periodically extracted for analysis using gas chromatography (Perkin Elmer Clarus 500 with flame ionization detector, using an Elite—1 (30 m, 0.32 mm) column with a helium carrier gas). To prepare SiO_2_ supported Au NPs 1.0 wt% [Au_9_(PPh_2_(p-tol))_8_](NO_3_)_3_ was deposited onto SiO_2_ spheres (Angstrom sphere monodispersed silica powder; 0.5 *μ*m particle size) using a wet impregnation method, followed by calcination at 300 °C leading to formation of ∼50 nm spherical gold particles on the surface of the support with a loading of 0.20 × 10^−4^ mmol Au/g of catalyst.

## 3. Results and discussion

### 3.1. Deposition of thin films of gold NPs

**Figure 1.**
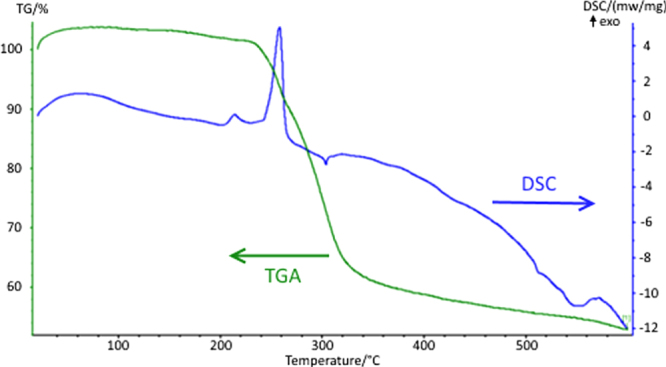
TGA and DSC data for [Au_9_(Ph_2_P(p-tol))_8_](NO_3_)_3_.

TGA (figure 1) showed a principle, relatively broad, weight loss between 250 and 340 °C of ∼40%, coincident with a large exotherm in the DSC trace. This corresponds well to the calculated weight loss (43.8%) for decomposition of [Au_9_(PPh_2_(p-tol))_8_](NO_3_)_3_ to gold only indicating it could be suitable for deposition of gold via AACVD.

**Figure 2.**
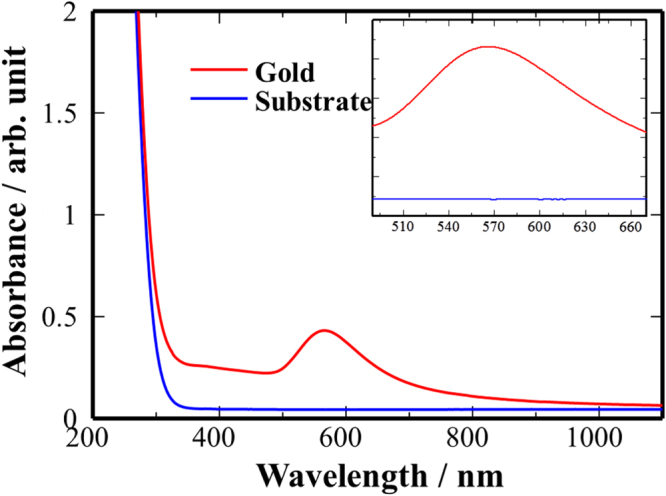
UV–vis spectrum of typical gold film deposited from [Au_9_(PPh_2_(p-tol))_8_](NO_3_)_3_ via AACVD.

Deposition of gold particles on glass substrates was achieved at 350 °C using a solution of [Au_9_(PPh_2_(p-tol))_8_](NO_3_)_3_ in methanol and the partially adherent films were transparent and red/purple in colour. UV–vis spectra (figure 2) displayed a broad absorbance between 500 and 650 nm, attributed to the surface plasmon resonance of the gold particles, with the peak centre at ∼566 nm.

Estimation of gold particle size from the peak absorbance of the plasmon observed using UV–vis spectroscopy (figure 2), using multipole scattering theory as described by Haiss *et al* [18] and assuming the gold particles were spherical and uncoated and the refractive index of the medium in contact with the particles was dominated by the glass substrate (1.517 at 546 nm), gave a value of 70 nm.

**Figure 3.**
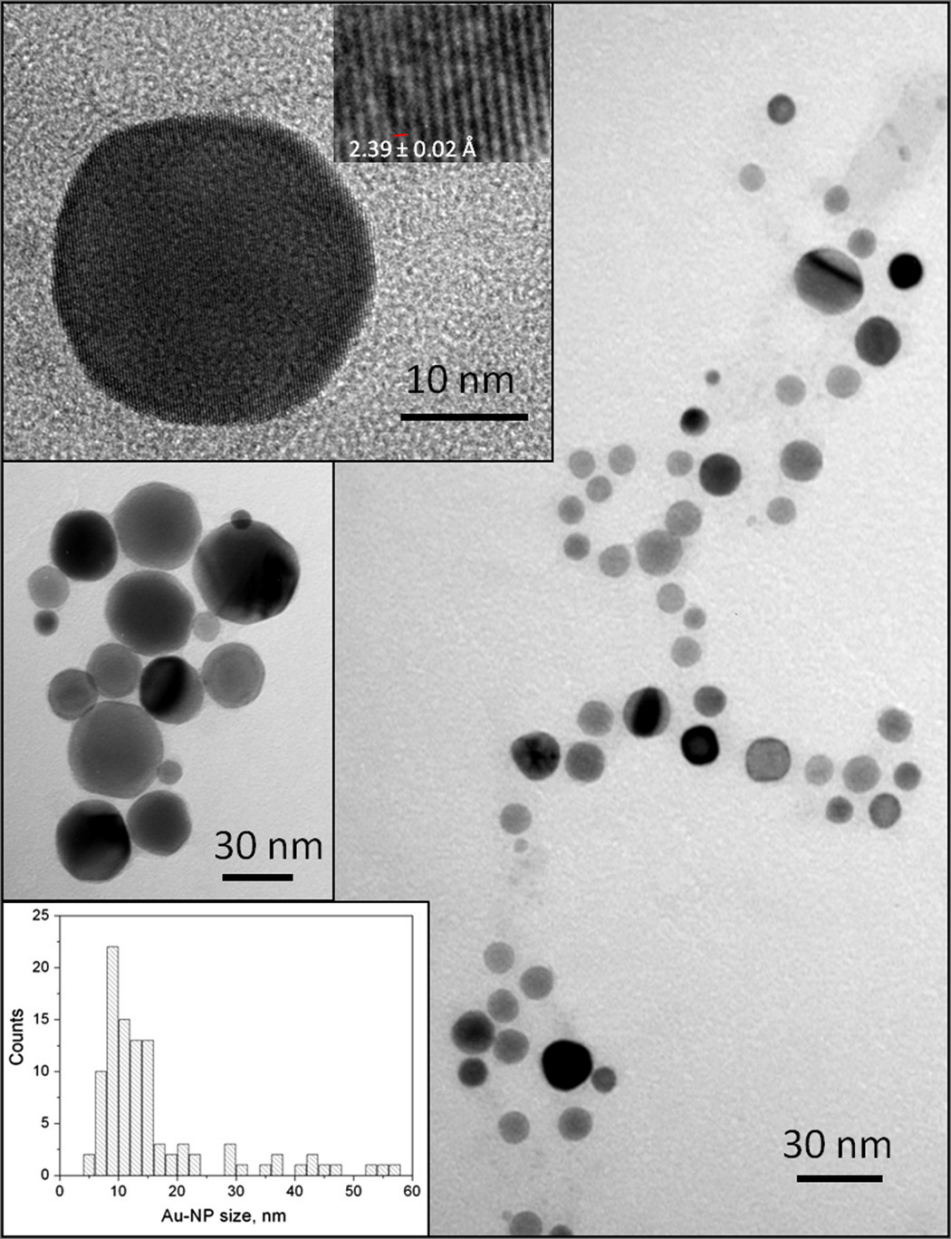
TEM images of gold particles removed from substrate for gold film deposited from [Au_9_(PPh_2_(p-tol))_8_](NO_3_)_3_ via AACVD. Bottom inset shows histogram of gold particle size distribution.

WDX of the gold coatings showed considerable carbon (∼19 at%) but no phosphorus contamination, suggesting the phosphine ligands were eliminated and the residual carbon was due to aerosol solvent pyrolysis. XRD revealed only peaks assigned to cubic gold (Fm-3m space group, *a* = *b* = *c* = 4.070(1) Å; ICDD card no. 00-04-0784 *a* = *b* = *c* = 4.0786 Å). SEM analysis showed the film to be composed of roughly spherical particles of diameter up to ∼50 nm diameter, which was confirmed by TEM (figure 3) with a wide dispersion of particle sizes observed between 5 nm and 58 nm (average = 16 ± 1 nm; *n* = 100). HRTEM images (figure 3 top and inset) of the gold particles exhibited highly ordered planar spacing of 2.39 ± 0.02 Å, consistent with the internal lattice spacing of the (111) plane of gold (*d* = 2.355 Å).

The size of these particles demonstrate that coalescence of the initial gold cluster cores (∼0.6 nm) [19] has occurred, although it is not possible to discern whether this is as a result of gas-phase reaction or aggregation on the substrate surface. Previous use of [Au_9_(PPh_3_)_8_](NO_3_)_3_ in solution-phase preparation of supported gold particles produced NP of ∼16 nm size [14], consistent with the average gold particle size found by TEM (16 ± 1 nm) for these AACVD deposited NP.

### 3.2. Co-deposition of thin films of gold NPs supported on tungsten oxide nanorods

**Figure 4.**
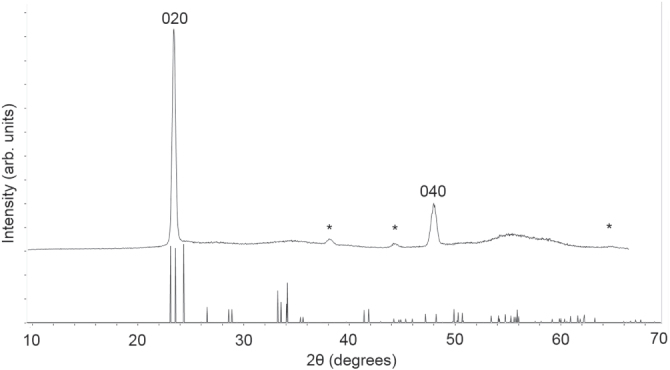
X-ray diffraction pattern of typical film co-deposited from W(OPh)_6_ and [Au_9_(PPh_2_(p-tol))_8_](NO_3_)_3_ via AACVD. The 〈020〉 and 〈040〉 diffraction peaks of monoclinic WO_3_ are labelled and peaks due to gold diffraction peaks are marked with asterisks.

Co-deposition of W(OPh)_6_ and [Au_9_(PPh_2_(p-tol))_8_](NO_3_)_3_ at 500 °C from acetone yielded blue adherent films of powdery appearance. XRD of the co-deposited films (figure 4) showed the presence of monoclinic tungsten oxide (P2_1_/n space group, ICDD card no. 72-0677 *a* = 7.306 00 Å, *b* = 7.540 00 Å, *c* = 7.692 00 Å and *β* = 90.88º) with preferred orientation in the [010] direction as seen previously from this tungsten precursor [9], with peaks attributable to gold also observed (Fm-3m space group, *a* = *b* = *c* = 4.07(6) Å; ICDD card no. 00-04-0784 *a* = *b* = *c* = 4.0786 Å).

WDX indicated a sub-stoichiometric WO_2.69_ composition, consistent with the blue colour of the films, with gold detected at ∼0.6 at% (Au/W atom ratio: ∼1/11 in precursors, ∼1/40 in film), phosphorus at <0.1 at% (P/W atom ratio: ∼1/13 in precursors, <1/500 in film), and carbon of ∼4 at% (C/W atom ratio: ∼40/1 in precursors, ∼1/7 in film). From these results we note the relatively poor incorporation of gold (∼25% efficiency), albeit higher than the 5–10% observed using inorganic precursors [10], and that the gold–phosphorus bonds are cleaved (P/Au ratio ∼1/1 in precursor, ∼1/17 in film). The dramatic reduction in carbon between precursor and film also indicates the precursors decompose as desired to eliminate unwanted contaminant elements under these conditions. XPS spectra revealed three W 4f_7/2_ environments observed at 34.9 eV (oxidation state +6, 66%), 35.8 eV (oxidation state +5, 22%) and 36.6 eV (variation in vacuum-level attributed to surface defects, 12%) indicating a WO_2.88_ stoichiometry [20]. A single Au 4f doublet was observed with Au 4f_7_ at 84.2 eV indicating the presence of metallic gold only and an Au/W ratio of ∼1/50, consistent with that found by WDX. No phosphorus was detectable indicating any contamination was below the detection limit of the instrument.

**Figure 5.**
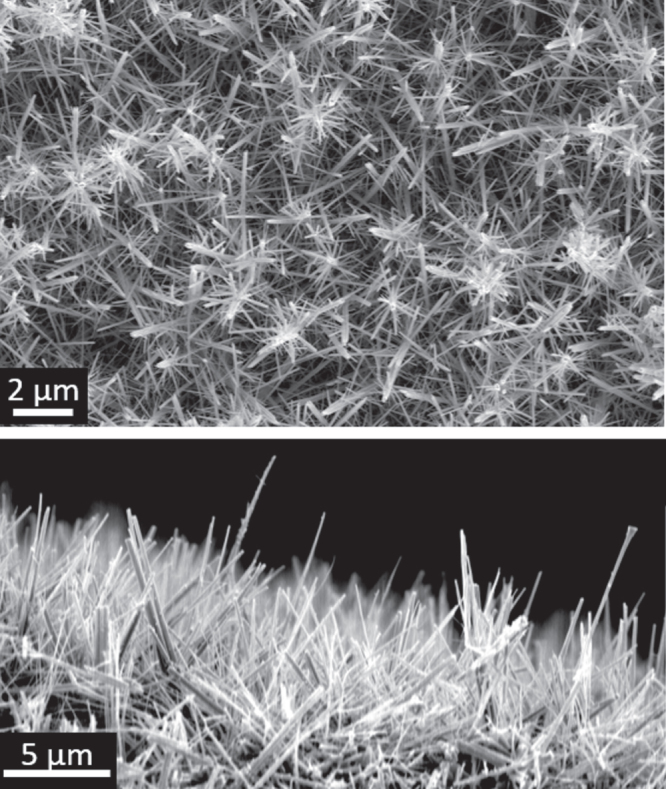
SEM images of a typical film co-deposited from W(OPh)_6_ and [Au_9_(PPh_2_(p-tol))_8_](NO_3_)_3_ via AACVD.

SEM showed the tungsten oxide had a nanorod morphology similar to that observed previously when depositing tungsten oxide alone under identical conditions [9], although some larger agglomerated structures were also observed. Films deposited both without and with (figure 5) the gold precursor present appeared similar in SEM images and no obvious gold particles were observed for films co-deposited with [Au_9_(PPh_2_(p-tol))_8_](NO_3_)_3_.

**Figure 6.**
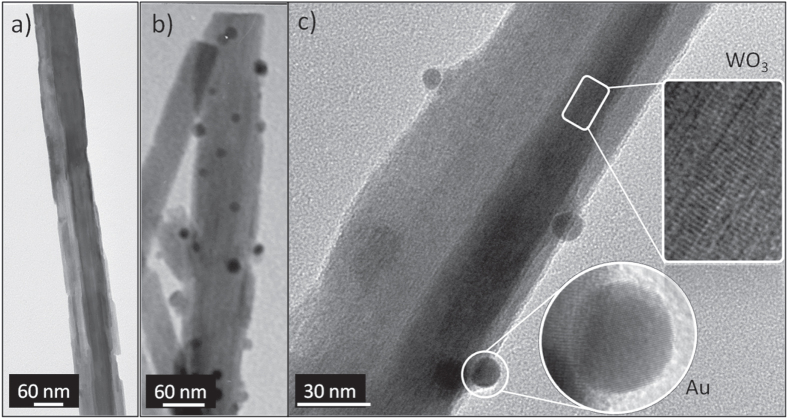
TEM images of particles removed from substrate after deposition via AACVD from; (a) W(OPh)_6_ only, (b) and (c) W(OPh)_6_ and [Au_9_(PPh_2_(p-tol))_8_](NO_3_)_3_.

In contrast TEM images clearly showed the presence of small (∼4–8 nm diameter) particles in co-deposited samples with the particles well distributed along the needle length (figure 6(b)). These particles are absent in the sample deposited without gold precursor (figure 6(a)). HRTEM images (figure 6(c)) exhibit highly ordered crystalline needle structures with marked planar spacing of 3.80 ± 0.04 Å, consistent with tungsten oxide ((001 or (010) planes) [10], with the decorating particles having planar spacing of 2.40 ± 0.03 Å, consistent with the (111) lattice spacing of gold. The gold particle sizes were on average slightly smaller and possessed a marginally narrower size distribution (4–11 nm diameter) than we observed using HAuCl_4_ as the gold precursor [10].

### 3.3. Catalytic testing of gold NPs supported on tungsten oxide nanorods and on SiO_2_ spheres

Table 1 shows the catalytic properties of the AACVD deposited samples, an SiO_2_ blank and an Au/SiO_2_ catalyst synthesized via solution-phase calcination/impregnation using the same gold precursor, which includes turnover numbers (TONs) for conversion of benzyl alcohol for either the total amount of catalyst (including support) or for the amount of gold only.

**Table 1.** Catalytic performance of tungsten oxide, gold and tungsten oxide supported gold films compared to silica supported gold particles produced using the same gold precursor via solution-phase synthesis.

Catalyst					Catalytic performance				
								Product selectivity (±2%)	
Cluster/support	AACVD solvent system	Process *T* (°C)	Mass catalyst (mg)	Mass gold (mg)	Conversion (±3%)	TON (catalyst)^a^	TON (gold only)^a^	Benz-aldehyde	Benzoic acid
Blank	N/A	N/A	N/A	N/A	5	—	—	68	32
WO_3_	Acetone	500	2	0	15	650	—	94	6
Au	Acetone	350	0.1	0.1	27	23 500	23 500	94	6
Au/WO_3_	Acetone	500	2	0.04	23	1000	50 000 (17 000)^c^	100	0
SiO_2_	N/A	N/A	200	0	5	2	—	75	25
Au/SiO_2_^b^	N/A	300^b^	200	8	53	7	580	97	3

a Turnonver number calculated based either on total mass of catalyst or mass of gold only.

b Synthesized via solution-phase impregnation/calcination.

c Value in brackets gives TON after subtraction of conversion due to tungsten oxide support.

The average mass of the co-deposited films was ∼2 mg, of which ∼1.8 wt% (as determined by WDX) was Au, i.e. ∼0.04 mg gold. The films deposited from the gold precursor alone had a mass of approximately 0.1 mg.

**Figure 7.**
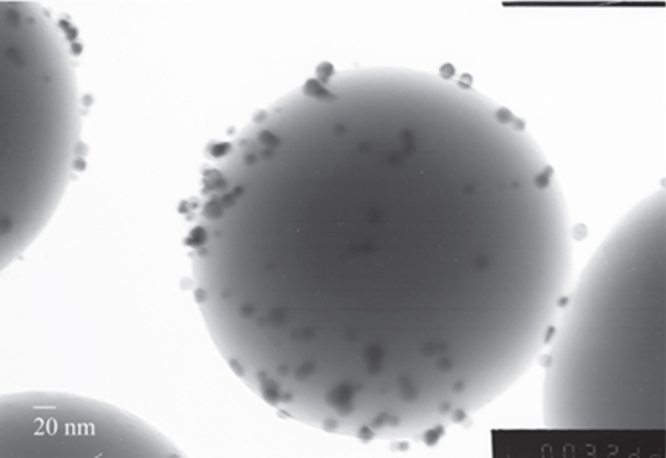
TEM image of gold particles deposited on SiO_2_ microspheres using wet impregnation/calcination.

Gold-NP supported on SiO_2_ (figure 7, 200 mg—equivalent to 8 mg Au), used as a standard for solution-phase synthesized material, gave 53% conversion of benzyl alcohol after 6 h with TON(gold) = 580. Tungsten oxide supported gold-NP (2 mg—equivalent to 0.04 mg Au) gave 23% conversion, however calculation of TON is complicated in this case as the tungsten oxide ‘support’ itself was also found to be active for benzyl alcohol oxidation (15% conversion, TON(catalyst) = 650). Tungsten oxides are versatile catalytic materials [21] and the oxidation of benzyl alcohol to benzaldehyde over tungsten oxide has been observed previously [22] with the oxidizing power of tungsten oxide attributed to the presence of oxygen defects and low valent tungsten centres [23], hence fully oxidized WO_3_ was found to be rather inactive. Therefore the activity found for the tungsten oxide support is consistent with the sub-stoichiometric composition as determined by WDX and XPS. It is worth noting that the activity of these tungsten oxide samples (TON(catalyst) = 650) is comparable to that of the gold-NP supported on SiO_2_ (TON(gold) = 580). Estimating the TON due to gold only for gold-NP supported on tungsten oxide, by subtraction of the conversion due to the support, gives TON(gold) = 17 000, whilst gold-NP deposited alone via AACVD were equally active with TON(gold) = 23 500, i.e. the CVD deposited gold-NP (either supported or ‘bare’) were an order of magnitude more active than gold-NP deposited from the same precursor via a solution phase method. It has been found previously that higher catalytic performance is observed in CVD deposited materials than those produced using solution-phase methods [24], although it should also be noted that the particle sizes (up to 50 nm) for the solution phase gold-NP were considerably larger than those for CVD deposited gold-NP (16 nm) or tungsten oxide supported gold-NP (∼6 nm).

The selective catalytic oxidation of hydrocarbons to their respective aldehydes is a major challenge for industry and reactions are frequently performed at very low conversion rates in order to avoid the formation of carboxylic acids [25]. Oxidation of benzyl alcohol to benzaldehyde is a useful test reaction as it is easily over-oxidized to benzoic acid and therefore provides a direct measure of the selectivity of the catalyst. Whilst both the SiO_2_ supported and AACVD deposited ‘bare’ gold-NP showed good selectivity to benzaldehyde (97% and 94% respectively) the tungsten oxide supported particles provided 100% selectivity. A support can act as a promoter to enhance the activity and selectivity of a reaction [26], for instance Pt-NPs supported on tungsten oxide exhibit excellent CO tolerance [27, 28] due to the presence of OH_ads_ on the tungsten oxide surface facilitating the oxidation of passivating CO_ads_ [29], hence the improvement in selectivity observed may be attributable to interaction between the tungsten oxide support and the gold NPs although further work, including *operando* studies, is on-going to prove this.

## 4. Conclusions

In summary the AACVD co-deposition of gold-NP and gold-NP supported on nanostructured tungsten oxide, utilizing a molecular gold cluster precursor, produces an extremely active catalyst compared to a traditionally prepared catalyst of gold NPs on microstructured silica using the same gold precursor. In addition increased selectivity in oxidation of benzyl alcohol to benzaldehyde is observed for AACVD co-deposited gold NP supported on tungsten oxide, attributed to interaction between the support and the metal NP. AACVD is readily adaptable to other nanostructured metal oxides and metal NPs and therefore this technique can be applied to a wide variety of not only catalytic materials, but also materials for use in other fields, such as plasmonic nanostructures and solar cells.
